# Neuroprotective Effects of Tuina in CP Rats Are Associated With Gut Microbiota Remodeling and Intestinal Barrier Restoration

**DOI:** 10.1002/brb3.71136

**Published:** 2025-12-17

**Authors:** Chenqin Si, Rui Qiao, Yu Liu, Ayipaxiaguli Kasimu, Danmei Chen, Lijian Xie, Ping‐ping Sun, Bing Li

**Affiliations:** ^1^ Department of Traditional Chinese Medicine Jinshan Hospital Affiliated to Fudan University Shanghai China; ^2^ Research Center for Clinical Medicine Jinshan Hospital Affiliated to Fudan University Shanghai China; ^3^ Yunnan University of Chinese Medicine Kunming China; ^4^ Department of Pediatrics Jinshan Hospital Affiliated to Fudan University Shanghai China; ^5^ School of Rehabilitation Science Shanghai University of Traditional Chinese Medicine Shanghai China; ^6^ Engineering Research Center of Traditional Chinese Medicine Intelligent Rehabilitation Ministry of Education Shanghai China

**Keywords:** cerebral palsy, gut microbiota, gut–brain axis, intestinal barrier

## Abstract

**Introduction:**

Cerebral palsy (CP) is a neurodevelopmental disorder that has been linked to gut microbiota dysbiosis. Although Tuina has shown neuroprotective effects, it remains unclear whether these benefits involve regulation of the gut–brain axis. This study aimed to evaluate the therapeutic effects of Tuina in CP rats, with emphasis on its potential regulation of the gut–brain axis.

**Methods:**

CP was induced in 7‐day‐old Sprague–Dawley rats through hypoxia–ischemia. Beginning on postnatal day 8 (P8), the Tuina group received daily Tuina therapy for 32 consecutive days. Motor function was assessed using the negative geotaxis test (P6–P12), the beam balance test (P36–P39), and the modified neurological severity score on P40. Gut microbiota composition was analyzed using 16S rRNA sequencing. Brain and intestinal histopathology were evaluated histologically via hematoxylin–eosin and Luxol fast blue staining. Protein expression of BDNF, Nrf2, GPX4, ZO‐1, and occludin was assessed via western blotting and immunofluorescence. Serum short‐chain fatty acids (SCFAs) were measured by mass spectrometry, whereas oxidative stress and intestinal barrier markers (superoxide dismutase, malondialdehyde, glutathione peroxidase, lipopolysaccharide [LPS], diamine oxidase [DAO], and D‐lactate [D‐LA]) were detected using enzyme‐linked immunosorbent assay.

**Results:**

In CP models induced by hypoxic–ischemic encephalopathy, significant brain injury and motor dysfunction were observed, accompanied by gut microbiota dysbiosis and impaired intestinal barrier function. Tuina intervention improved motor function and growth, regulated gut microbiota, and increased serum SCFA levels. It also enhanced intestinal barrier proteins (occludin, ZO‐1), reduced serum levels of LPS, DAO, and D‐LA, and increased the expression of brain‐derived BDNF, Nrf2, and GPX4.

**Conclusion:**

Tuina significantly alleviated brain injury and improved motor function in CP rats. These effects were associated with modulation of the gut microbiota and restoration of intestinal barrier integrity, suggesting that the gut–brain axis may mediate the neuroprotective effects of Tuina.

## Graphical abstract

TN‐treated CP rats show improved gut microbiota composition and enhanced intestinal barrier function, accompanied by increased serum SCFAs, elevated BDNF levels in the brain, activation of the NRF2/GPX4 antioxidant pathway, and reduced brain inflammatory markers, including IL‐6 and TNF‐α.

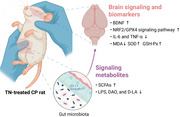

## 1 Introduction

Cerebral palsy (CP), a neurodevelopmental condition caused by nonprogressive brain injury, is clinically characterized by motor dysfunction, often accompanied by growth delay as well as cognitive and emotional impairments (Wimalasundera and Stevenson 2016; Uday et al. 2017). Typical neuropathologic features include periventricular leukomalacia, cerebral atrophy, and cortical dysplasia (O'Shea 2008). Increasing evidence suggests that the gut–brain axis plays a critical role in the pathophysiology of CP (Huang et al. 2021; Peng et al. 2023; Chu et al. 2024). The gut–brain axis is a complex bidirectional communication network spanning the immune system, neuroendocrine system, enteric nervous system, systemic circulation, and vagus nerve (Ullah et al. 2023). This multisystem crosstalk is mediated by microbiota‐derived metabolites, neuroactive compounds, and hormones that collectively regulate brain function and neural homeostasis (Carloni and Rescigno 2023). Emerging evidence shows marked differences in gut microbiota composition between children with CP and healthy controls, including reduced beneficial taxa and increased potentially pathogenic microorganisms (Huang et al. 2019). Such dysbiosis can impair the intestinal barrier, increase permeability (“leaky gut”), exacerbate neuroinflammation, and worsen neurological injury (Dargenio et al. 2023; Wu et al. 2025). Accordingly, the gut–brain axis likely plays a pivotal role in neurological recovery and represents a promising therapeutic avenue and target for CP intervention.

Tuina, a traditional Chinese manual therapy, is widely used in the management of children with CP because of its established safety profile and noninvasive nature. In clinical practice, Tuina improves cognitive and motor functions, reduces disability rates, and enhances quality of life in children with CP (Eliasson et al. 2018; C. Zhang et al. 2021). Mechanistically, many animal studies indicate that Tuina promotes growth and development and improves learning, memory, and motor function in CP rats. These effects may involve attenuation of cerebral inflammation and NLRP3‐mediated pyroptosis (Niu et al. 2021), inhibition of endothelial PARthanatos to preserve blood–brain barrier integrity (Kong et al. 2025), modulation of 5‐HT signaling and synaptic proteins to enhance synaptic plasticity (Xu et al. 2022), and reshaping of epigenetic regulation related to neurodevelopment and inflammation (Y. Zhang et al. 2019). Notably, the benefits of Tuina may extend beyond direct effects on the central nervous system. In murine models of colitis, Tuina alleviates inflammation, restores colonic structure, and increases microbial diversity (H. Wang et al. 2022), suggesting that it can modulate gut homeostasis. Nevertheless, whether the beneficial effects of Tuina in CP involve regulation of the gut–brain axis remains unknown.

Building on our previous study (Qiao et al. 2023), we hypothesized that the neuroprotective effects of Tuina in CP rats are associated with gut microbiota remodeling and intestinal barrier restoration via the gut–brain axis. To test this hypothesis, this study aimed to investigate the therapeutic potential of Tuina and its regulatory effects on the gut–brain axis using CP rat models.

## 2 Materials and Methods

### 2.1 Animals

Male Sprague–Dawley rats (7 days old, weighing 20–30 g) were obtained from Shanghai Jihui Laboratory Animal Co., Ltd. (Shanghai, China) and housed at the Laboratory Animal Center of the Shanghai Public Health Clinical Center. The pups were nursed by their dams and maintained under standard laboratory conditions: temperature 22–27°C, relative humidity 50%–70%, and a 12‐h light/dark cycle, with ad libitum access to standard chow and water. All experimental procedures were performed in accordance with the ethical guidelines approved by the Experimental Animal Ethics Committee of the Shanghai Public Health Clinical Center (approval No. 2022‐A004‐01).

### 2.2 Model Establishment

On postnatal day 7 (P7), pups were anesthetized with 5% isoflurane. The left common carotid artery was carefully exposed and ligated. After confirming hemostasis, the skin incision was sutured and disinfected. Following recovery from anesthesia, the pups were placed in a hypoxic chamber at 37°C containing 92% nitrogen (N_2_) and 8% oxygen (O_2_) for 3 h. After hypoxic exposure, the pups were returned to their dams for regular feeding.

### 2.3 Experimental Design and Tuina Therapy Intervention

**FIGURE 1:**
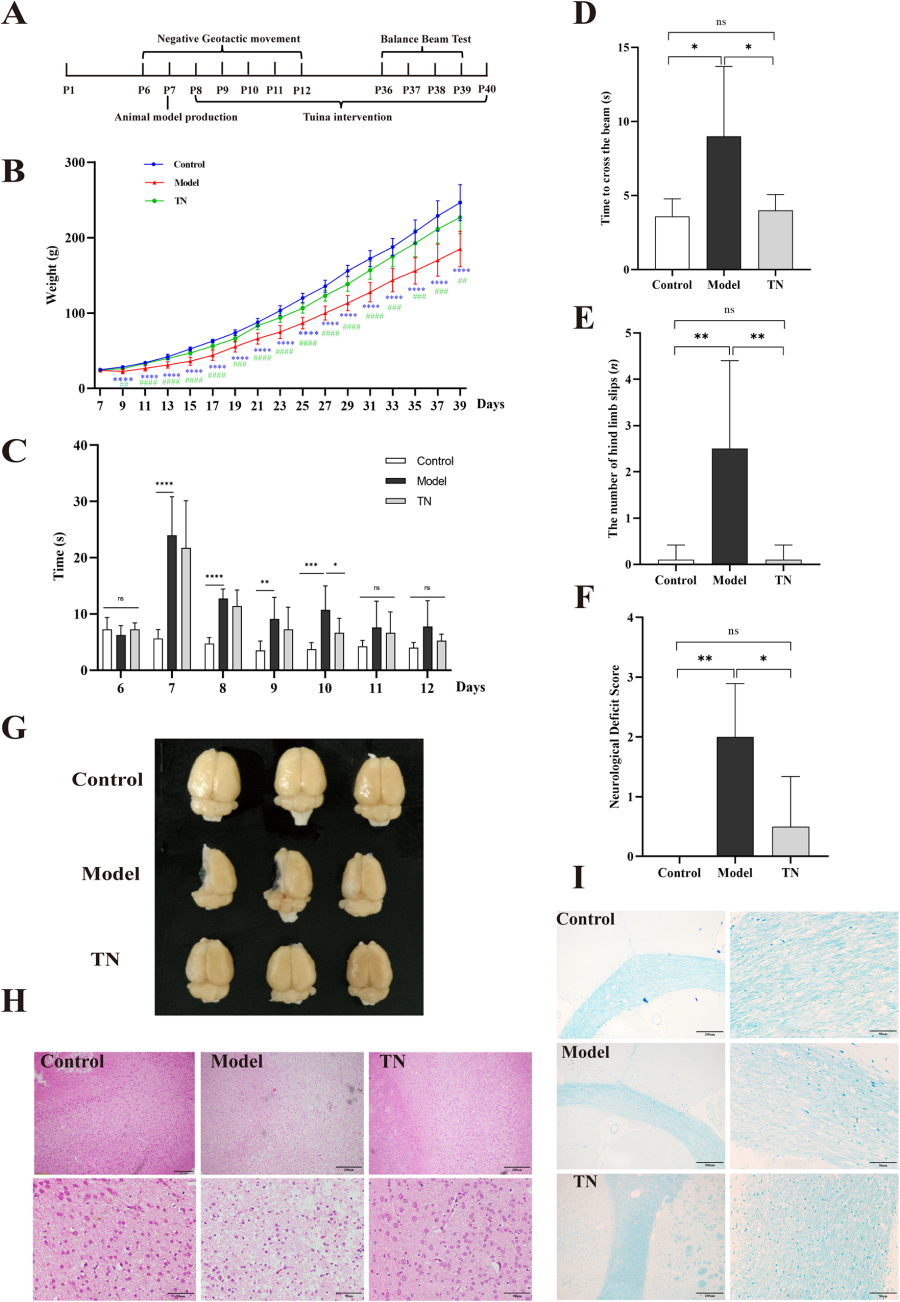
Tuina promotes growth and motor function recovery in CP rats. (A) Schematic diagram of the experimental design. (B) Body weight changes in each group of rats. Data are presented as mean ± SD (*N* = 8). The asterisk (*) indicates a significant difference versus the control group; the has (#) indicates a significant difference versus the TN group. *p < 0.05, **p < 0.01, ***p < 0.001. (C) Results of the negative geotaxis test for each group. Data are presented as mean ± SD (*N* = 8). (D–F) Time to cross the balance beam (D), number of hind limb slips (E), and neurological function scores (F) for each group. Data are presented as mean ± SD (*N* = 8). (G) Gross brain injury examination in each group of rats. (H) Histological analysis of the cortex using HE staining. Top panels: low‐magnification images (scale bar: 200 µm); bottom panels: high‐magnification images (scale bar: 50 µm). (I) Histological analysis of the cortex using LFB staining. Left panels: low‐magnification images (scale bar: 200 µm); right panels: high‐magnification images (scale bar: 50 µm). *p < 0.05, **p < 0.01, ***p < 0.001, ****p < 0.0001. CP, cerebral palsy; HE, hematoxylin–eosin; LFB, Luxol fast blue; P, postnatal; SD, standard deviation; TN, Tuina.

The experimental procedure is illustrated in Figure 1A. The study spanned from P1 to P40. On P6, pups were randomly assigned to three groups: control, model, and Tuina. On P7, pups in the control group underwent a sham operation, whereas those in the model and Tuina groups underwent induction of the CP model. Behavioral assessments included the negative geotaxis test, conducted from P6 to P12, and daily body weight measurements, from P7 to P40. The balance beam test was performed from P36 to P39. On P40, the modified neurological severity score (mNSS) was assessed, and tissue samples were collected for further analysis. Tuina intervention was administered to the Tuina group starting on P8 for 32 consecutive days. Each session lasted 15 min, with a massage frequency of 120 strokes per minute. The intervention followed a specific sequence: (1) spinal manipulation, (2) limb manipulation, and (3) abdominal rubbing. The specific procedures were performed in accordance with previously established protocols (Qiao et al. 2023).

### 2.4 Gut Microbiota Analysis

Fecal samples were collected aseptically, immediately flash‐frozen in liquid nitrogen, and stored at −80°C until further analysis. Gut microbiota profiling was performed using 16S rRNA gene sequencing. Total DNA was extracted from fecal samples, and the V3–V4 region of the 16S rRNA gene was amplified using specific primers. The PCR products were purified, quantified, and sequenced on the Illumina MiSeq platform using paired‐end reads. Sequencing data were processed and analyzed using QIIME2 software, including quality filtering, operational taxonomic unit clustering, and diversity analysis. Taxonomic annotation was performed against the Greengenes database.

### 2.5 Behavioral Test

The negative geotaxis test was performed on rats in all three groups on P6‒P12. Each rat was placed in a head‐down position on an inclined plane set at a 45° angle, and the time required for the rat to reorient from the head‐down to the head‐up position was recorded.

The balance beam test was conducted to evaluate motor coordination and balance in young rats. A wooden beam measuring 1 cm in width and 50 cm in length, elevated 30 cm above the surface, was used. Each rat was placed at one end of the beam and encouraged to traverse to the opposite end. The time required to cross the beam and the number of slips (defined as any instance of a paw slipping off the beam) were recorded. Prior to the formal test, rats underwent a 3‐day training period (P36–P38) to acclimate to the apparatus. The formal test was conducted on the fourth day (P39), during which each rat completed three trials at 30‐min intervals. The average crossing time and the mean number of slips were calculated for each rat as indices of motor coordination and balance.

### 2.6 mNSS

On P40, an mNSS assessment was performed to evaluate neurological function. The mNSS criteria are summarized in Table S1, with higher scores indicating more severe neurological deficits.

### 2.7 Serum Short‐Chain Fatty Acid (SCFA) Detection

A 100‐µL plasma sample was collected from each experimental group. Triethylammonium bicarbonate (TEAB, 4.5 µL, 1 M) and 8 M urea buffer solutions were prepared, and alkylation was performed by adding iodoacetamide (5 µL, 375 mM) to each sample. The samples were then washed with acetone (660 µL) and incubated overnight at 37°C. Subsequently, samples from each group were labeled using the iTRAQ kit. The labeled samples were mixed in equal volumes, desalted, and lyophilized. The dried samples were reconstituted in a pH 10 solution containing 2% acetonitrile and centrifuged. High‐performance liquid chromatography separation was performed using a C18 column maintained at 45°C. After vacuum drying, the eluates were dissolved in buffer and fractionated using the Dionex NCS3500 system, followed by analysis via nano‐electrospray ionization tandem mass spectrometry (nano‐ESI–MS/MS).

### 2.8 Western Bolt Analysis

Total protein was extracted from the samples, and the concentration was determined and normalized accordingly. Equal amounts of protein were separated by sodium dodecyl sulfate–polyacrylamide gel electrophoresis and subsequently transferred onto polyvinylidene difluoride membranes. The membranes were blocked at room temperature for 1 h in TBST buffer containing 5% nonfat milk. Following blocking, the membranes were incubated overnight at 4°C with specific primary antibodies: Nrf2 (1:2000, Abcam, ab31163), BDNF (1:2000, Abcam, ab108319), GPX4 (1:2000, Abcam, ab125066), ZO‐1 (1:5000, Bioss, ab‐4023R), occludin (1:2000, Bioss, bs‐1001R), and β‐actin (1:2000, CST, 4970S). After three washes with TBST, the membranes were incubated with HRP‐conjugated secondary antibodies (anti‐rabbit, 1:5000, CST, 7074S) at room temperature for 2 h. Protein bands were visualized using an enhanced chemiluminescence kit (Merck Millipore) and imaged with a ChemiDoc MP system (Bio‐Rad). Densitometric analysis was performed using ImageJ software.

### 2.9 Immunofluorescence (IF) Staining

Paraffin‐embedded sections were dried at 60°C for 2 h and sequentially treated with xylene, absolute ethanol, graded ethanol, and distilled water. Antigen retrieval was performed by boiling the sections in diluted 50x EDTA solution, allowing them to cool to room temperature, and subsequently washing them with PBS. The sections were blocked with 10% serum for 10 min at room temperature, followed by overnight incubation at 4°C with primary antibodies diluted at ratios of 1:100–1:500 (Nrf2, 1:200, Abcam, ab31163; BDNF, 1:200, Abcam, ab108319; GPX4, 1:200, Abcam, ab125066; ZO‐1, 1:200, Bioss, ab‐34023R; occludin, 1:500, Bioss, bs‐10011R; IL‐6, 1:200, Abcam, ab9324; TNF‐α, 1:200, Abcam, ab6671). The following day, the sections were washed with PBS and incubated with fluorescent secondary antibodies for 45 min. After additional PBS washes, the sections were counterstained with DAPI for nuclear visualization and imaged using a confocal microscope.

### 2.10 Histological Examination

After drying the paraffin sections at 60°C for 2 h, they were sequentially treated with xylene, absolute ethanol, graded ethanol, and distilled water. For hematoxylin and eosin (H&E) staining, the sections were immersed in hematoxylin for 15 min, differentiated in acid alcohol, blued in weak ammonia water, and stained with 0.5% aqueous eosin for 10 min. Subsequently, the sections were dehydrated through a graded ethanol series, cleared in xylene, and mounted with neutral resin. For Luxol fast blue (LFB) staining, the sections were incubated in LFB solution for 20 h, rinsed with 95% ethanol to remove excess dye, differentiated with lithium carbonate, counterstained with eosin, dehydrated through graded ethanol, cleared in xylene, and mounted with neutral resin. Finally, all stained sections were examined and photographed under a microscope for analysis.

### 2.11 Enzyme‑Linked Immunosorbent Assay (ELISA)

Serum samples were analyzed using ELISA kits purchased from Shanghai Enzyme‐linked Biotechnology Co., Ltd. The following kits were used in this study: Rat Lipopolysaccharide (LPS) ELISA Kit (Catalog No. ml003197V), Rat Diamine Oxidase (DAO) ELISA Detection Kit (Catalog No. ml003420V), Rat D‐Lactic Acid (D‐LA) ELISA Kit (Catalog No. ml273710V), Rat Superoxide Dismutase (SOD) ELISA Kit (Catalog No. ml077379V), Rat Malondialdehyde (MDA) ELISA Kit (Catalog No. ml077384V), and Rat Glutathione Peroxidase (GSH‐Px) ELISA Kit (Catalog No. ml097316V).

### 2.12 Statistical Analysis

Statistical analyses were performed using SPSS version 25.0 (Chicago, IL, USA). Data are presented as mean ± standard deviation (SD). The normality of data distribution was confirmed using the Shapiro‒Wilk test, and the homogeneity of variances was verified using Levene's test. Group differences were analyzed using one‐way analysis of variance (ANOVA), followed by post hoc tests. Specifically, when variances were homogeneous, the Least Significant Difference test was applied; otherwise, Dunnett's T3 test was used. A significance level of α = 0.05 was used, and *p* < 0.05 was considered statistically significant. Graphs were generated using GraphPad Prism 9.5.

## 3 Result

### 3.1 Effects of Tuina on Body Weight and Brain Tissue Damage in CP Rats

To evaluate the effects of Tuina on the growth and development of CP rats, body weight was monitored across all groups. Although body weight gradually increased in each group, rats in the model group exhibited a significantly slower rate of weight gain than those in the control and Tuina groups, with significant differences observed from P9 to P39 (Figure 1B). Brain injury was then assessed in CP rats. Gross examination revealed that the control group had an intact and symmetrical bilateral cerebral cortex, whereas the model group exhibited marked damage, including cortical atrophy and liquefaction. The Tuina group showed only mild atrophy without obvious defects (Figure 1G). H&E staining (Figure 1H) showed that cortical neurons in the control group were numerous, were densely arranged, and exhibited normal nuclei with no obvious pathological changes. In contrast, the injured cortex in the model group contained many swollen, atrophic, and ruptured neurons, with irregular cell sizes, increased staining intensity, and abundant cellular debris. Neuronal density was reduced, and the cells were disorganized, with decreased cytoplasmic volume. Following Tuina intervention, brain tissue injury was markedly attenuated, with fewer necrotic neurons and a more organized, compact neuronal arrangement. Similarly, LFB staining revealed that the Tuina group had less demyelination and vacuolation than the model group (Figure 1I). Collectively, these findings indicate that Tuina promotes the growth and development of CP rats and significantly reduces brain tissue injury.

### 3.2 Effects of Tuina on Motor Function in CP Rats

To evaluate the therapeutic effects of Tuina on motor dysfunction, CP rats were subjected to a series of behavioral tests. In the negative geotaxis test (Figure 1C), no significant differences were observed between groups at baseline (P6). After model induction (P7–P10), the model group exhibited significantly longer response times than the control group. Notably, the consistently shorter response times in the Tuina group than in the model group, which did not reach statistical significance at P8 and P9, became statistically significant by P10. Further assessment with the balance beam test revealed significantly shorter crossing times and fewer foot slips in the Tuina group than in the model group (Figure 1D,E). Consistent with these findings, the Tuina group also demonstrated significantly better neurological function scores (Figure 1F). Collectively, these results indicate that Tuina significantly improves motor function in CP rats.

### 3.3 Effects of Tuina on the Gut Microbiota and SCFAs in CP Rats

**FIGURE 2:**
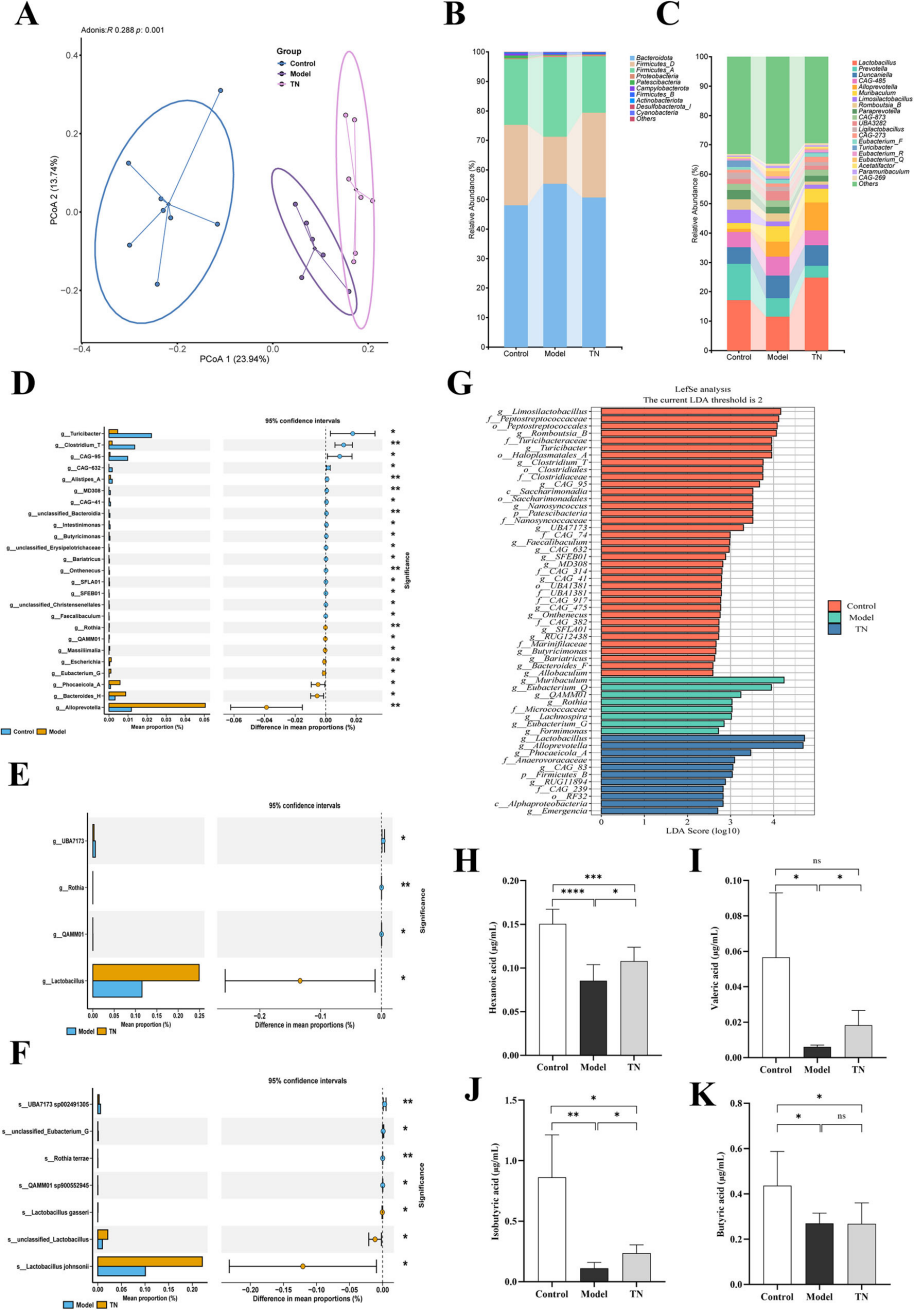
Tuina reshaped the gut microbiota of CP rats and promoted the production of SCFAs. (A) β‐Diversity analysis based on PCoA. (B, C) Relative abundance of the top 10 genera (B) and top 20 species (C) in the gut microbiota of each group. (D–F) STAMP analysis of the differential gut microbiota using Welch's *t*‐test: (D) at the genus level between the control and model groups, (E) at the genus level between the model and Tuina groups, and (F) at the species level between the model and Tuina groups. (G) LEfSe analysis of the gut microbiota between the three groups, with an LDA score >2 and *p* < 0.05. (H–K) Plasma concentrations of hexanoic (H), valeric (I), isobutyric (J), and butyric (K) acids. Data are presented as mean ± SD (*N* = 6). *p < 0.05, **p < 0.01, ***p < 0.001, ****p < 0.0001. PCoA, principal coordinates analysis; LEfSe, LDA effect size; SCFAs, short‐chain fatty acids; SD, standard deviation.

To determine whether Tuina ameliorates gut microbiota dysbiosis in CP rats, we performed 16S rRNA gene sequencing on fecal samples from all groups. β‐Diversity analysis based on principal coordinates analysis (PCoA) revealed distinct clustering of the gut microbiota between the control, model, and Tuina groups (Figure 2A). Furthermore, STAMP and LEfSe analyses identified significant differences in microbial community composition between groups (Figure 2D–G). Collectively, these results indicate that CP induction caused marked microbial dysbiosis, which was substantially ameliorated by Tuina intervention. At the phylum level, the model group showed a characteristic decrease in Firmicutes and an increase in Bacteroidetes, resulting in an altered Firmicutes/Bacteroidetes ratio. Notably, Tuina intervention effectively reversed this dysbiosis (Figure 2B). Analysis of the top 20 genera showed that *Lactobacillus* was most abundant in the Tuina group, exceeding its levels in both the control and model groups (Figure 2C). STAMP and LEfSe analyses further corroborated these findings, demonstrating that *Lactobacillus*—including *L. gasseri*, *L. johnsonii*, and unclassified *Lactobacillus* taxa—was significantly enriched in the Tuina group. In contrast, genera, such as Rothia and QAMM01, were specifically enriched in the model group (Figure 2E–G).

Given the established role of gut microbiota‐derived SCFAs in regulating central nervous system function, we hypothesized that the observed microbial shifts may influence SCFA levels. Serum analyses supported this hypothesis: the model group showed significantly reduced concentrations of hexanoic, isobutyric, butyric, and valeric acids. Tuina intervention partially restored hexanoic, isobutyric, and valeric acid levels, although it had only a limited effect on butyric acid (Figure 2H–K). In summary, these findings suggest that Tuina restores gut microbiota homeostasis in CP rats, markedly increases the relative abundance of *Lactobacillus*, and promotes the production of SCFAs.

### 3.4 Tuina Alleviates Intestinal Tissue Pathology and Inflammation in CP Rats

**FIGURE 3:**
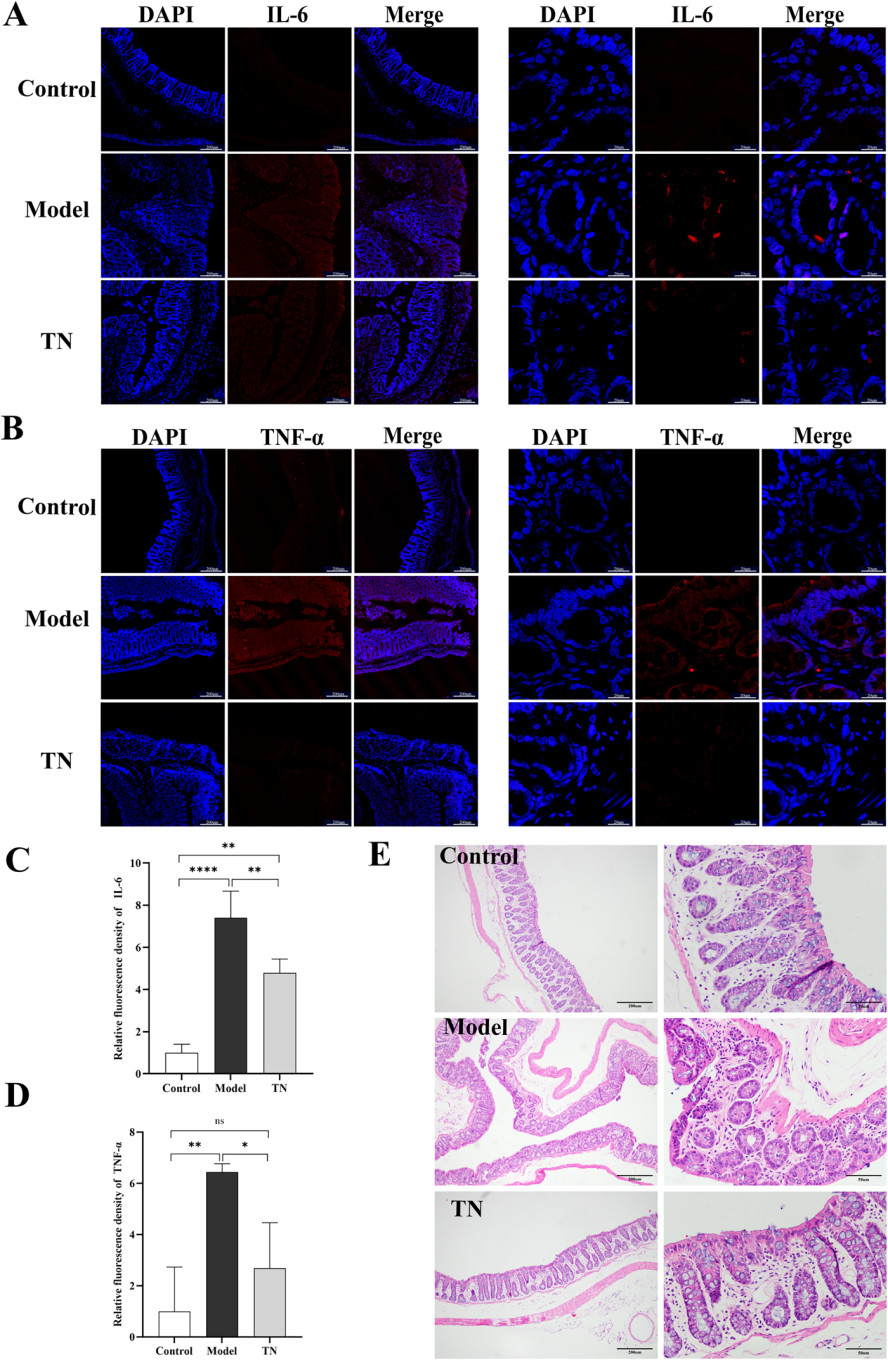
Tuina reduces pathological damage and inflammatory responses in the intestines of CP rats. (A, B) Immunofluorescence staining of IL‐6 (A) and TNF‐α (B) in intestinal tissues. Left panels: low‐magnification images (scale bar: 200 µm); right panels: high‐magnification images (scale bar: 20 µm). (C, D) Quantification of mean fluorescence intensity for IL‐6 (C) and TNF‐α (D). Data are presented as mean ± SD (*N* = 3). (E) HE staining of intestinal tissues: left panels show low‐magnification images (scale bar: 200 µm), and right panels show high‐magnification images (scale bar: 50 µm). *p < 0.05, **p < 0.01, ***p < 0.001, ****p < 0.0001. TNF‐α, tumor necrosis factor‐alpha; IL‐6, interleukin‐6; HE, hematoxylin–eosin; SD, standard deviation.

We further evaluated the effects of Tuina on intestinal health in CP rats. HE staining revealed pronounced inflammatory lesions in the model group, characterized by disruption of the mucosal architecture, reduced numbers of glands, and goblet cell atrophy. These pathological alterations were markedly ameliorated following Tuina treatment (Figure 3E). In addition, IF staining showed that the fluorescence intensities of the proinflammatory cytokines IL‐6 and TNF‐α in intestinal tissues were significantly lower in the Tuina group than in the model group (Figure 3A,B), a finding further confirmed by quantitative analysis (Figure 3C,D). Collectively, these findings indicate that Tuina effectively attenuates intestinal pathological injury and inflammatory responses in CP rats.

### 3.5 Effects of Tuina on Intestinal Barrier Function and Plasma Levels of LPS, DAO, and D‐LA in CP Rats

**FIGURE 4:**
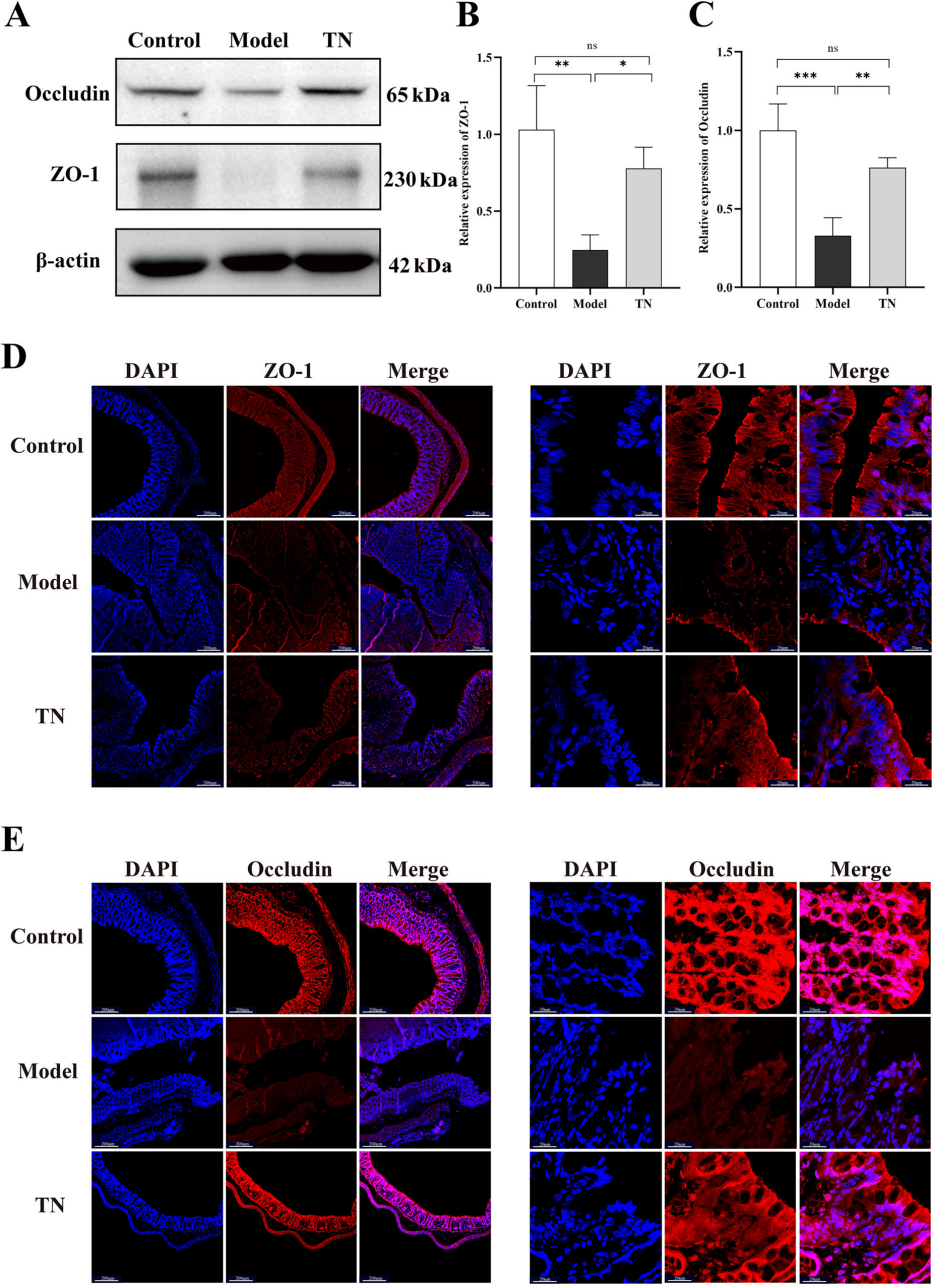
Tuina alleviates intestinal barrier dysfunction in CP rats. (A) Western blot analysis of ZO‐1 and occludin protein expression in intestinal tissues of each group. (B, C) Quantification of relative protein levels of ZO‐1 (B) and occludin (C) based on western blot analysis. Data are presented as mean ± SD (*N* = 3). (D, E) Immunofluorescence staining of ZO‐1 (D) and occludin (E) in intestinal tissues of each group. Left panels: low‐magnification images (scale bar: 200 µm); right panels: high‐magnification images (scale bar: 20 µm). *p < 0.05, **p < 0.01, ***p < 0.001, ****p < 0.0001. ZO‐1, zonula occludens‐1; SD, standard deviation; CP, cerebral palsy.

**FIGURE 5:**
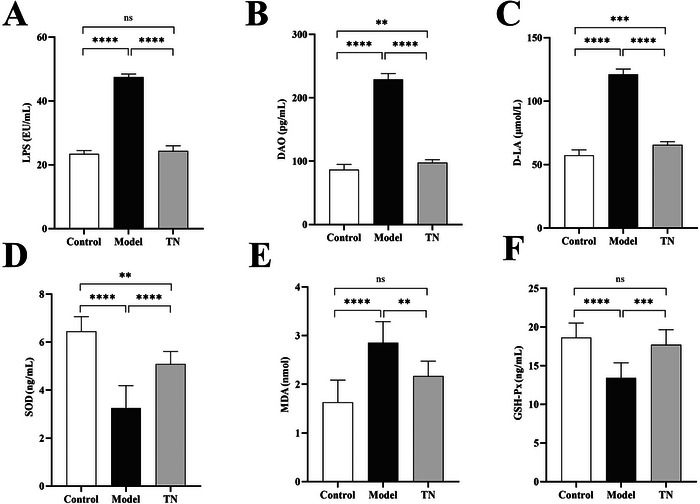
Effects of Tuina on LPS, DAO, D‐LA, SOD, MDA, and GSH levels in CP rats. (A–C) Plasma concentrations of LPS (A), DAO (B), and D‐LA (C) in each group. Data are presented as mean ± SD (*N* = 8). (D–F) Plasma concentrations of SOD (D), MDA (E), and GSH‐Px (F) in each group. Data are presented as mean ± SD (*N* = 8). *p < 0.05, **p < 0.01, ***p < 0.001, ****p < 0.0001. LPS, lipopolysaccharide; DAO, diamine oxidase; D‐LA, D‐lactic acid; MDA, malondialdehyde; SOD, superoxide dismutase; GSH‐Px, glutathione peroxidase; SD, standard deviation; CP, cerebral palsy.

We further evaluated the effects of Tuina on intestinal barrier integrity in CP rats. Western blotting and IF analyses showed that Tuina significantly upregulated the expression of the tight junction proteins ZO‐1 and occludin (Figure 4). In parallel, Tuina treatment significantly reduced plasma levels of the gut permeability markers LPS, DAO, and D‐LA (Figure 5A–C), and attenuated systemic oxidative stress by decreasing MDA levels and increasing the activities of SOD and GSH‐Px (Figure 5D–F). These results indicate that Tuina improves intestinal function in CP rats by strengthening barrier integrity, reducing permeability, and mitigating oxidative stress.

### 3.6 Effects of Tuina on Brain Inflammation and BDNF Expression in CP Rats

**FIGURE 6:**
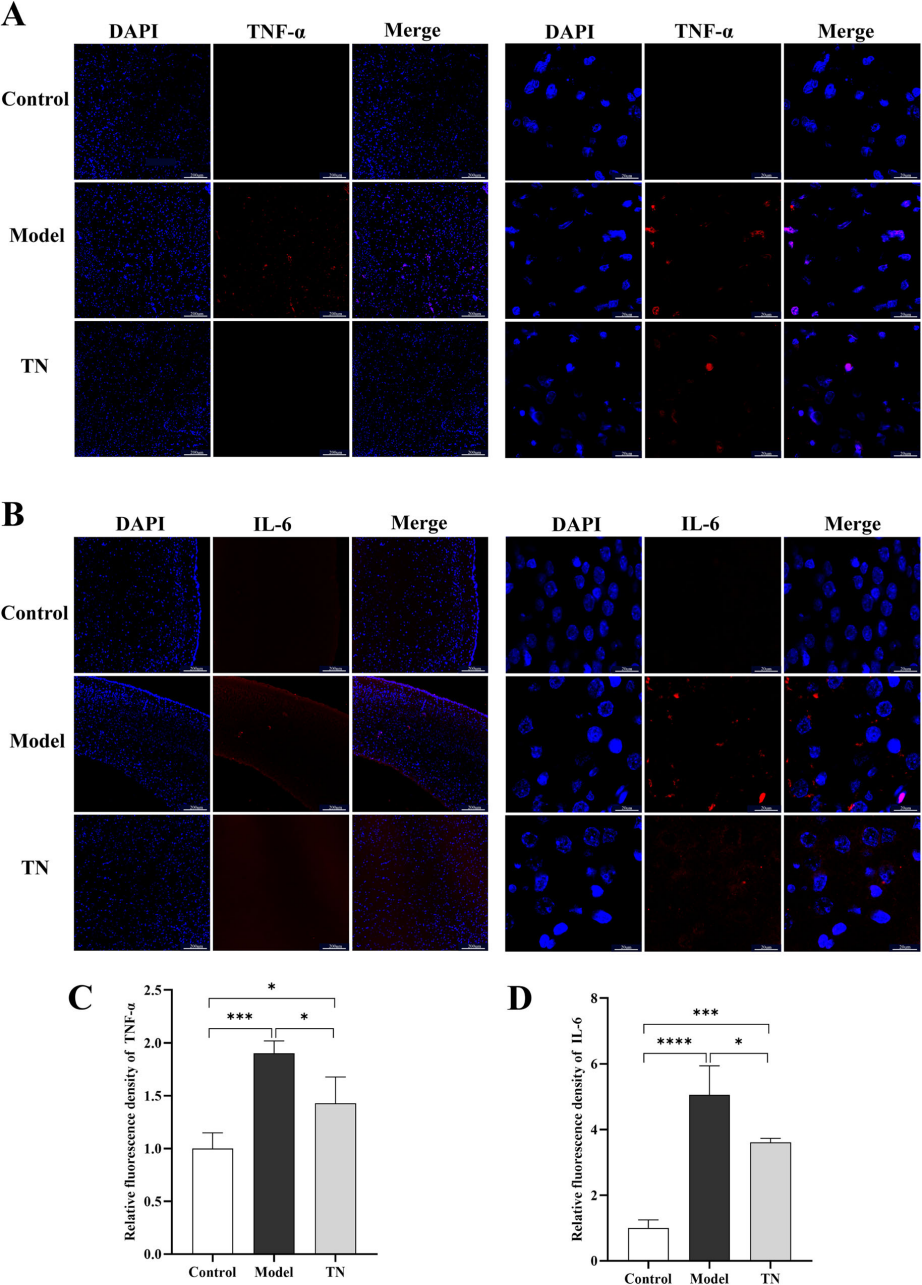
Tuina alleviates neuroinflammation in the brain of CP rats. (A, B) Immunofluorescence staining of TNF‐α (A) and IL‐6 (B) in the cortex of each group. Left panels: low‐magnification images (scale bar: 200 µm); right panels: high‐magnification images (scale bar: 20 µm). (C, D) Quantification of mean fluorescence intensity for IL‐6 (C) and TNF‐α (D). Data are presented as mean ± SD (*N* = 3). *p < 0.05, **p < 0.01, ***p < 0.001, ****p < 0.0001. TNF‐α, tumor necrosis factor‐alpha; IL‐6, interleukin‐6; SD, standard deviation; CP, cerebral palsy.

**FIGURE 7:**
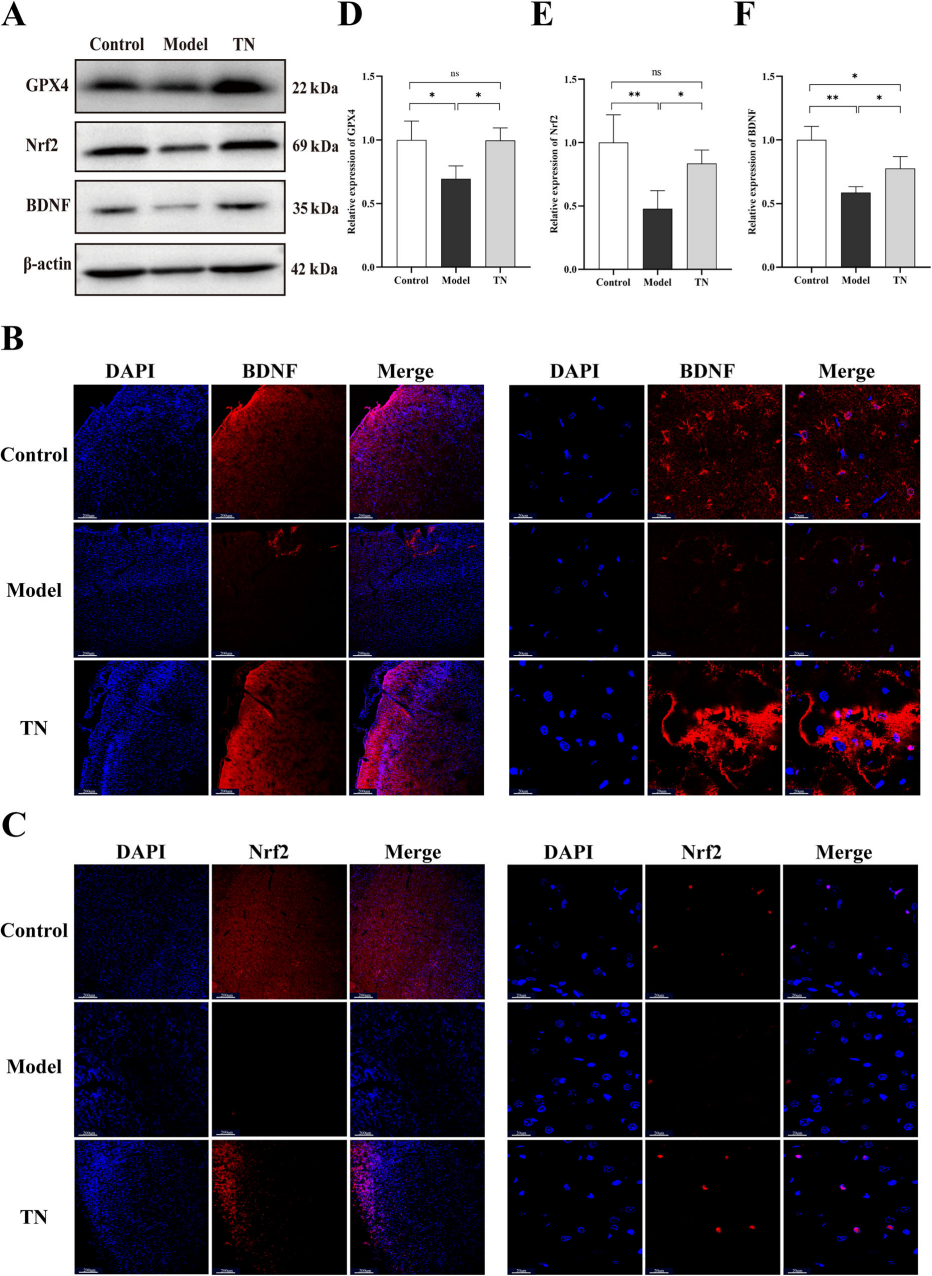
Tuina promotes BDNF expression and activates the Nrf2/GPX4 pathway in the brains of CP rats. (A) Western blot analysis of BDNF, Nrf2, and GPX4 protein expression in the cortex of each group. (B, C) Immunofluorescence staining of BDNF (B) and Nrf2 (C) in the cortex of each group. Left panels: low‐magnification images (scale bar: 200 µm); right panels: high‐magnification images (scale bar: 20 µm). (D–F) Quantification of relative protein levels of GPX4 (D), Nrf2 (E), and BDNF (F) based on western blot. Data are presented as mean ± SD (*N* = 3). *p < 0.05, **p < 0.01, ***p < 0.001, ****p < 0.0001. BDNF, brain‐derived neurotrophic factor; Nrf2, nuclear factor erythroid 2‐related factor 2; GPX4, glutathione peroxidase 4; SD, standard deviation; CP, cerebral palsy.

Given that SCFAs are key mediators of gut–brain axis signaling with important effects on the nervous system, we examined cortical molecules associated with SCFA activity, including the proinflammatory cytokines IL‐6 and TNF‐α, neurotrophic factor BDNF, and antioxidant proteins Nrf2 and its downstream effector GPX4. The cortical fluorescence intensities of IL‐6 and TNF‐α were significantly higher in the model group than in the Tuina group (Figure 6). In addition, cortical BDNF expression was markedly reduced in the model group but significantly increased in the Tuina group (Figure 7A,B,F). Western blot analysis further demonstrated that Tuina markedly increased cortical expression of Nrf2 and GPX4 in CP rats (Figure 7A,D,E). Consistent with these results, IF staining confirmed a pronounced upregulation of Nrf2 in the Tuina group (Figure 7C). These findings suggest that SCFAs may mediate the attenuation of neuroinflammation, the regulation of neurotrophic factor expression, and the improvement of oxidative stress in the brain.

## 4 Discussion

This study demonstrates that Tuina therapy exerts neuroprotective effects in CP rats, as evidenced by improved motor coordination, enhanced growth, and reduced histopathological brain damage. Importantly, our findings suggest that these beneficial effects may be mediated, at least in part, through modulation of the gut microbiota and restoration of intestinal barrier integrity, implicating the gut–brain axis as a potential mechanistic pathway underlying the therapeutic action of Tuina.

In this study, we successfully established a CP rat model that exhibited typical motor dysfunction as well as neuronal and myelin injury. Tuina intervention promoted weight gain, improved motor coordination and balance, and attenuated histological brain damage. These findings are consistent with those of previous reports on the neuroprotective effects of Tuina (Niu et al. 2021; Chen et al. 2025), which predominantly focused on brain‐specific improvements after treatment. However, the potential systemic mechanisms underlying these central effects, including possible interactions along the gut–brain axis, have rarely been explored. In contrast to prior studies, we therefore extended our investigation to examine the effects of Tuina on gut microbiota composition and intestinal barrier function in CP rats, aiming to explore whether the gut–brain axis contributes to its therapeutic effects.

The gut–brain axis constitutes a complex bidirectional communication network integrating neural, endocrine, immune, and metabolic pathways (Q. Wang, Yang, et al. 2023). Accumulating evidence indicates that disturbances within this axis contribute to the pathogenesis of various neurodevelopmental and neurodegenerative disorders, including autism spectrum disorder, Alzheimer's disease, and stroke (You et al. 2024; Jabbari Shiadeh et al. 2025). Gut dysbiosis and compromised intestinal barrier integrity promote systemic inflammation and neuroinflammatory responses through translocation of bacterial metabolites and immune mediators (Mishra et al. 2025). Consistent with clinical reports of gastrointestinal dysfunction and chronic inflammation in children with CP (Huang et al. 2019; Peng et al. 2023), our CP rats exhibited significant microbial imbalance and intestinal barrier disruption. Tuina intervention effectively reversed these abnormalities, as evidenced by increased abundance of beneficial taxa, decreased pro‐inflammatory cytokine levels, reduced systemic oxidative stress, and restored expression of tight‐junction proteins. These findings suggest that the gut–brain axis may represent a key pathway through which Tuina exerts its therapeutic effects. Mechanistically, Tuina may influence vagal afferent activity as well as autonomic and immune regulation through peripheral somatosensory stimulation, similar to that induced by acupuncture (Ulloa et al. 2017; M. Wang, Liu, et al. 2023; Yao et al. 2025), thereby modulating gut motility, secretion, and local immune responses. This bidirectional neural regulation may contribute to the restoration of intestinal homeostasis and, consequently, improve brain function via the gut–brain axis.

One of the most notable findings of this study was the marked enrichment of *Lactobacillus* species following Tuina treatment. *Lactobacillus*, a well‐established commensal genus, supports microbial homeostasis, inhibits the growth of harmful bacteria, suppresses intestinal inflammation, and enhances mucosal barrier function (Maske et al. 2021; Bernard et al. 2023; Li et al. 2023; Yin et al. 2023; Di Chiano et al. 2024). Our data extend these observations by demonstrating that Tuina, a nonpharmacological intervention, can promote the endogenous enrichment of *Lactobacillus* while simultaneously inhibiting the growth of harmful bacteria, including members of *Porphyromonadaceae*, *Rothia*, and *Clostridium*. Consistent with previous reports, these pathogenic taxa are closely associated with intestinal inflammation and related pathological changes, particularly under conditions of immune suppression or barrier dysfunction (Uzal et al. 2022; Troci et al. 2024; Maccioni et al. n.d.). Importantly, *Lactobacillus* species are important contributors to SCFA production through cross‐feeding interactions with other gut bacteria (Moens et al. 2017; Bui et al. 2019; Tan et al. 2023). SCFAs serve as critical signaling molecules within the gut–brain axis (O'Riordan et al. 2022), reinforcing intestinal and blood–brain barrier integrity by upregulating tight junction proteins such as claudin‐5, occludin, and ZO‐1, thereby limiting translocation of inflammatory mediators (Ma et al. 2022; Fock and Parnova 2023). SCFAs also activate G protein–coupled receptors, suppress NF‐κB–dependent inflammatory signaling, and modulate microglial activation in the central nervous system (Du et al. 2024). Moreover, butyrate and propionate function as histone deacetylase inhibitors, promoting transcriptional activation of neurotrophic and antioxidant genes, including BDNF and Nrf2 (Jaworska et al. 2019; He et al. 2022). This epigenetic mechanism enhances synaptic plasticity and cellular resilience to oxidative stress. In this study, Tuina‐treated rats exhibited significantly elevated circulating SCFA levels, accompanied by upregulation of BDNF, Nrf2, and GPX4 expression in the brain—key mediators of neuronal plasticity and antioxidant defense. These findings support the notion that the neuroprotective effects of Tuina may be associated with enhanced microbial production of SCFAs, which in turn could activate antioxidant and neuroprotective pathways via the gut–brain axis.

This study had some limitations. Although we observed significant gut microbiota alterations following Tuina intervention that correlated with functional improvements, the experimental design does not establish causality. The observed microbial changes could result directly from Tuina or may arise secondarily from overall systemic improvement. Future studies employing more direct approaches, such as fecal microbiota transplantation from Tuina‐treated rats into germ‐free or antibiotic‐treated CP model recipients, would be invaluable to conclusively determine whether gut microbiota modulation is a direct and necessary mechanism underlying the therapeutic effects of Tuina. However, implementing such approaches in neonatal rodents poses substantial challenges, including ethical constraints and technical complexity.

## 5 Conclusion

In summary, Tuina therapy markedly attenuates brain injury, promotes growth, and improves motor function in CP rats. These beneficial effects are closely associated with the restoration of intestinal homeostasis and elevated systemic levels of SCFAs. By activating the BDNF and Nrf2/GPX4 pathways, SCFAs—important gut‐derived metabolites—may facilitate neural repair and antioxidant defense via the gut–brain axis. Collectively, our findings provide preliminary evidence supporting a potential mechanism through which Tuina therapy exerts neuroprotective effects in CP.

## Author Contributions

**Chenqin Si**: conceptualization, methodology, formal analysis, investigation, data curation, writing – original draft, writing – review and editing, visualization. **Rui Qiao**: conceptualization, methodology, formal analysis, investigation, data curation. **Yu Liu**: investigation. **Ayipaxiaguli Kasimu**: investigation. **Danmei Chen**: writing – original draft, funding acquisition. **Ping‐ping Sun**: supervision. **Lijian Xie**: supervision, funding acquisition. **Bing Li**: conceptualization, supervision, project administration, funding acquisition.

## Funding

This research was supported by the National Natural Science Foundation of China (grant numbers: 82174522 and 82305428), the Seventh Period Jinyin Medical Key Specialty A‐level Pediatrics (JSZK2023A04), and the Medical Key Disciplines of Shanghai (2024ZDXK0056).

## Ethics Statement

All experimental procedures were conducted in accordance with the ethical guidelines approved by the Experimental Animal Ethics Committee of the Shanghai Public Health Clinical Center (Approval number: 2022‐A004‐01).

## Consent

Written informed consent for publication was obtained from all participants.

## Conflicts of Interest

The authors declare no conflicts of interest.

## Supporting information




**Supplementary Table**: brb371136‐supp‐0001‐TableS1.docx

## Data Availability

The authors declare that all other data supporting the findings of this study are available within the article and its Supporting Information, or are available from the authors upon request.
