# Supplementary material for: Neuroprotective Effects of Tuina in CP Rats Are Associated With Gut Microbiota Remodeling and Intestinal Barrier Restoration
Source: Brain Behav. 2025 Dec 17;15(12):e71136. doi: 10.1002/brb3.71136 (PMC12710083; doi:10.1002/brb3.71136)
Supplement: Supplementary file 1 — Supplementary Table: brb371136‐supp‐0001‐TableS1.docx [file BRB3-15-e71136-s001.docx]

**Table S1:** Modified Neurological Severity Score

| **Category** | \| **Response** \| \| --- \| | \| **Score** \| \| --- \| |
| --- | --- | --- | --- | --- |
| **General Deficit** | Consciousness normal | 0 |
|  | Lethargic | 1 |
|  | Coma / No response | 2 |
| **Eye Stimulation** | Normal eyelid response | 0 |
| \|  \| \| --- \| | No eye response to pain stimulation | 1 |
|  | Unilateral avoidance | 1 |
|  | Bilateral avoidance | 2 |
| **Respiration** | Normal | 0 |
|  | Weak | 2 |
| **Movement** | Normal | 0 |
|  | Weak, immobile, tilting towards injured side | 1 |
|  | Severe tilting towards injured side | 2 |
| **Limb Withdrawal** | Quick | 0 |
|  | Weakened | 1 |
|  | No response | 2 |
